# A Formative Cross‐Sectional Study to Assess the Knowledge, Attitude, and Practices of Caregivers of Children Under Five Towards Malaria Control in the Buea Health District of Cameroon

**DOI:** 10.1155/jotm/5566468

**Published:** 2026-06-30

**Authors:** Mildred Nje Laban, Elvis Asangbeng Tanue, Emmanuel Yenshu Vubo, Dickson Shey Nsagha

**Affiliations:** ^1^ Department of Public Health and Hygiene, Faculty of Health Sciences, University of Buea, P.O Box 63, Buea, Cameroon, ubuea.cm; ^2^ Department of Sociology and Anthropology, Faculty of Social and Management Sciences, University of Buea, P.O Box 63, Buea, Cameroon, ubuea.cm

**Keywords:** attitudes, Buea Health District, Cameroon, caregivers, knowledge, malaria, practices, prevention, under-five children

## Abstract

**Background:**

Malaria is a major public health problem in Cameroon, disproportionately affecting children under five. The success of prevention and control programmes depends on caregivers’ knowledge, attitudes, and practices (KAP). Assessing these domains and their determinants could provide evidence for targeted interventions. The study set out to determine the levels of KAP towards malaria prevention and identify associated factors among caregivers of children under five in the Buea Health District in Cameroon.

**Methods:**

A community‐based cross‐sectional study was conducted among 394 caregivers in peri‐urban and rural communities of the Buea Health District. The survey was conducted from April 3 to July 12, 2024. Participants were interviewed using a structured questionnaire which captured the KAP of caregivers of under‐five children. Association between variables and community setting was measured using chi square test, and a multivariate logistic regression model was fitted to identify factors associated with adequate knowledge, positive attitudes, and good practices on malaria prevention.

**Results:**

Most respondents were female (85.3%), parents of the child (88.6%), and self‐employed (78.2%). Overall, 58.4% (95% CI: 53.5%–63.0%) had adequate knowledge, 42.9% (95% CI: 38.0%–47.8%) demonstrated positive attitudes, and 54.1% (95% CI: 49.2%–59.0%) reported good practices towards malaria prevention. Adequate knowledge was associated with being a caregiver rather than household head (AOR = 2.23, 95% CI: 1.10–4.49), employed (AOR = 8.12, 95% CI: 1.86–35.28), and living in brick houses (AOR = 1.88, 95% CI: 1.20–2.95).

Positive attitudes were more likely among caregivers in peri‐urban areas (AOR = 1.72, 95% CI: 1.12–2.65), those with secondary (AOR = 2.21, 95% CI: 1.04–4.67) or tertiary education (AOR = 3.18, 95% CI: 1.16–8.71), and those with better living standards (e.g., owned a television set, radio, or phone [AOR = 2.16, 95% CI: 1.29–3.63]). Good practices were associated with peri‐urban residence (AOR = 1.73, 95% CI: 1.12–2.67), adequate knowledge (AOR = 2.06, 95% CI: 1.37–3.09), positive attitudes (AOR = 1.84, 95% CI: 1.23–2.77), higher maternal education (secondary: AOR = 1.90, 95% CI: 1.01–3.58), and requesting malaria testing before treatment (AOR = 3.15, 95% CI: 2.01–4.94).

**Conclusion:**

Over half of caregivers had adequate knowledge and good practices, but fewer demonstrated positive attitudes, with rural caregivers showing significant gaps. KAP were influenced by sociodemographic, socioeconomic, and behavioural factors. Targeted health education, particularly for rural caregivers and those with lower education or fewer assets, could improve malaria prevention behaviours and reduce disease burden among children under five.

## 1. Background

Malaria remains one of the leading causes of morbidity and mortality worldwide, with the heaviest toll borne by sub‐Saharan Africa (SSA). According to data from the World Malaria Report of 2024, an estimated 249 million malaria cases and 608,000 deaths occurred globally in 2023, with the World Health Organization (WHO) African Region accounting for 94.0% of cases and 95.0% of deaths [1]. Children under five years represented 76.0% of malaria deaths in the region, underscoring their extreme vulnerability [1]. Cameroon is among the world’s 15 highest‐burden malaria countries, contributing 2.6% of global cases and 2.0% of deaths in 2023, with an estimated 7.3 million cases and 11,600 deaths [1]. National prevalence among children under five has increased from 24.0% in 2018 to 26.1% in 2022 [2]. Malaria remains a leading cause of health service use, accounting for approximately 30.0% of consultations, 44.0% of hospitalizations, and 18% of facility‐based deaths, with 41.0% of under‐five deaths attributed to the disease [2, 3]. The Buea Health District (BHD), located in the Mount Cameroon area, experiences perennial malaria transmission driven by a subtropical highland climate and favourable breeding conditions for vectors [4, 5]. Socioeconomic factors such as low educational attainment, poor housing, and limited access to healthcare exacerbate the burden, particularly among rural residents, pregnant women, and children under five.

Caregivers play a pivotal role in malaria prevention for young children, including the use of long‐lasting insecticidal nets (LLINs), timely care‐seeking, environmental hygiene, and adherence to treatment. However, previous studies in Cameroon and other endemic countries have shown that adequate knowledge does not always translate into positive attitudes or sustained preventive practices [6–8]. Misconceptions, cost barriers, cultural beliefs, and inconsistent usage of LLINs persist, undermining the effectiveness of national control measures.

Formative research on caregivers’ knowledge, attitudes, and practices (KAP) is essential to design targeted context‐appropriate interventions. One promising, scalable strategy is the use of mobile health (mHealth) interventions. Mobile phone short‐message service (SMS) allows for the delivery of timely, consistent, and culturally tailored health education directly to caregivers, overcoming geographic and infrastructural barriers. Evidence from other low‐resource settings shows that SMS‐based interventions can significantly improve maternal and child health behaviours, including malaria prevention practices [9, 10]. This study aims at providing a formative cross‐sectional assessment of caregivers’ KAP towards malaria prevention in the BHD, with the ultimate goal of informing targeted mHealth strategies to strengthen malaria control and accelerate progress towards Cameroon’s malaria elimination targets.

## 2. Materials and Methods

### 2.1. Study Area

This study was conducted in peri‐urban and rural communities of the BHD in Fako Division of the South West Region of Cameroon. The BHD lies on the eastern slopes of Mount Cameroon and has a subtropical highland climate with perennial transmission, peaking during the rainy season and at lower altitudes. Data were collected in six communities selected to capture ecological and service‐access contrasts: the rural communities of Tole, Bova, and Bokwaongo, and the peri‐urban communities of Buea Town, Sandpit, and Muea. Across these sites, small bushes, households surrounded with vegetation, and seasonal stagnant water create breeding habitats for *Anopheles* vectors. Housing is predominantly a mix of cement‐brick and wooden/plank structures, with implications for indoor mosquito entry. Malaria in the area is mainly due to *Plasmodium falciparum*, with transmission sustained year‐round and intensifying during rains.

### 2.2. Study Design

This was a community‐based cross‐sectional study which was conducted between April 3 and July 12, 2024. Caregiver’s KAP on malaria prevention were recorded using structured questionnaires to obtain baseline data for a context‐specific design of health education intervention to improve childhood malaria control.

### 2.3. Study Population, Inclusion Criteria, and Exclusion Criteria

The study population was composed of caregivers in households with children under‐five years.

We included caregivers of children under five who have been resident in the community for at least 6 months before the commencement of data collection. Caregivers who were unable to follow the questionnaire due to illness or disabilities that prevented them from understanding the questions were excluded from data collection.

### 2.4. Sample Size Determination

The sample size for the cross‐sectional survey was calculated using the Cochran formula:

(1) Sample size, n=Z2p1−pd2

where *n* = minimum sample size, *z* = 1.96 at a 95% CI, *d* = desired precision (5%), and *p* = 52.9% (proportion of caregivers of children under five with adequate knowledge on malaria prevention [11].

(2) n=1.962x 0.52910.5291−/0.052=383 caregivers of children under−five.

However, a total of 394 caregivers of children under five were enrolled into the study.

### 2.5. Sampling and Recruitment of Participants

A list of all peri‐urban and rural communities in the BHD was generated, from which three peri‐urban communities (Buea Town, Sandpit, and Muea) and three rural communities (Tole, Bova, and Bokwaongo) were randomly selected by balloting. Within each selected community, community health workers (CHWs) accompanied the Research Assistants to identify households with at least one child under‐five years. The CHWs guided the team to eligible households, and recruitment continued sequentially until the target sample size for that community was attained. At each household, the study objective was explained, eligibility was confirmed, and informed consent was obtained from the caregiver before enrolment into the study.

### 2.6. Data Collection

A structured questionnaire with closed‐ended questions was developed following a comprehensive literature review of malaria KAP studies. The full questionnaire is provided as Supporting Information 1. The tool was designed to capture information on caregivers’ sociodemographic and economic characteristics, environmental and housing conditions, knowledge of malaria transmission, recognition of symptoms, prevention methods, and attitudes and practices towards malaria control. Trained research assistants administered the questionnaire in English or Pidgin English during face‐to‐face interviews. Before field implementation, the tool was pretested in a nonstudy community to assess clarity, cultural appropriateness, and comprehension. Feedback from the pretest was used to refine the wording and adapt the questionnaire for the local context. Quality control measures included daily reviews of completed Kobo Collect submissions by research team, observation of at least one interview per enumerator each day, and verification of completeness and consistency of responses.

### 2.7. Statistical Analysis

Completed Kobo Collect forms were exported to Microsoft Excel for cleaning and coding, then imported into the Statistical package for Social Sciences version 25.0 for analysis. Descriptive statistics were used to summarize sociodemographic characteristics and responses for the KAP. KAP scores were calculated by summing the points for all relevant items. For single‐response questions, correct answers were assigned 1 point; for multiple‐response questions, each correct option was weighted so that the total possible score for that question equalled 1 point. For each domain of the KAP, scores were converted into percentages, and participants were classified as having adequate knowledge, positive attitudes, or good practices if their score was equal to or greater than the overall mean for that domain. Bivariate analysis using the Chi‐square test was performed to assess associations between sociodemographic factors and KAP outcomes. Variables with *p* < 0.05 in the bivariate analysis were included in a multivariate logistic regression model to identify independent predictors, with adjusted odds ratios (AOR) and 95% confidence intervals (CI) reported. Statistical significance was set at *p* < 0.05.

### 2.8. Ethical Considerations

Ethical approval was obtained from the Institutional Review Board of the Faculty of Health Sciences, University of Buea (reference number: 2024/1975‐02/UB/SG/IRB/FHS). Administrative authorization was obtained from the South West Regional Delegation of Public Health and from the Buea District Health Service. Written informed consent was obtained from all participants before enrolment, and confidentiality was ensured by using unique identification codes for each participant.

## 3. Results

### 3.1. Sociodemographic Characteristics of Caregivers of Children Under Five

A total of 394 caregivers took part in the study. The majority, 336 (85.3%) of the respondents were females. Participants were mainly [318 (80.7%)] the primary caregiver of the children. More than half [199 (58.2%)] of the fathers of the children were aged between 31 and 40 years, whereas their mothers were mostly between 21 and 30 years (173; 45.4%). The majority, 349 (88.6%) of the respondents were the parents of the children under five. The fathers and mothers of the children had mostly completed secondary level of education (251; 63.7% and 254; 64.5%), respectively (Table 1).

**TABLE 1:** Sociodemographic characteristics of caregivers of children under five in the Buea Health District of Cameroon.

Variable	Category	Frequency (%)
Sex of respondent	Female	336 (85.3)
	Male	58 (14.7)
	Total	394 (100)

Respondent’s relationship with the child	Caregiver/head of household	34 (8.6)
	Caregiver	318 (80.7)
	Head of household	42 (10.7)
	Total	394 (100)

Age group of father (years)	< 21	2 (0.6)
	21–30	62 (18.1)
	31–40	199 (58.2)
	> 40	79 (23.1)
	Total	342 (100)

Age group of mother (years)	< 21	19 (5.0)
	21–30	173 (45.4)
	31–40	173 (45.4)
	> 40	16 (4.2)
	Total	381 (100)

Religion of household	Muslim	12 (3.0)
	None	10 (2.5)
	Pentecostal	120 (30.5)
	Protestant	175 (44.4)
	Roman Catholic	77 (19.5)
	Total	394 (100)

Relationship of respondent with the under fives in the household	Son/daughter	349 (88.6)
	Grandchild	27 (6.9)
	Brother/sister’s child	13 (3.3)
	Others	5 (1.3)
	Total	394 (100)

Highest completed level of education of father	None	6 (1.5)
	Not applicable	37 (9.4)
	Primary	58 (14.7)
	Secondary	251 (63.7)
	Tertiary	42 (10.7)
	Total	394 (100)

Highest completed level of education of mother	None	5 (1.3)
	Not applicable	9 (2.3)
	Primary	84 (21.3)
	Secondary	254 (64.5)
	Tertiary	42 (10.7)
	Total	394 (100)

### 3.2. Socioeconomic Characteristics of Caregivers of Children Under Five

The majority, 308 (78.2%) of the caregivers were self‐employed committed to agriculture and small businesses as their main source of income. The participants mostly reported earning less than 50,000 FCFA (about 85 USD) as their household’s monthly income (282; 71.6%). Most (248; 62.9%) of the households were housing between four and six persons. The majority, 343 (87.1%) of the households had a television and almost all participants (379; 96.2%) owned mobile phones. More than half (232; 58.9%) of the household were constructed using planks and mainly (322; 81.7%) used cement on the floor (Table 2).

**TABLE 2:** Socioeconomic characteristics of caregivers of children under five in the Buea Health District of Cameroon.

Variable	Category	Frequency (%)
Main occupation	Employed	49 (12.4)
	Housewife	10 (2.5)
	Self‐employed	308 (78.2)
	Student	13 (3.3)
	Unemployed	14 (3.6)
	Total	394 (100)

Household’s estimated monthly income (FCFA)	< 50,000	282 (71.6)
	50–100,000	93 (23.6)
	> 100,000	19 (4.8)
	Total	394 (100)

Number of persons living in household	1–3	53 (13.5)
	4–6	248 (62.9)
	6–8	61 (15.5)
	> 8	32 (8.1)
	Total	394 (100)

Type of asset owned by household	Radio	102 (25.5)
	Television	343 (87.1)
	Mobile phone	379 (96.2)

Type of material used for house construction	Brick	162 (41.1)
	Plank	232 (58.9)
	Total	394 (100)

Type of floor in the house	Cement	322 (81.7)
	Tiles	57 (14.5)
	Mud	15 (3.8)
	Total	394 (100)

### 3.3. Knowledge Towards Malaria Prevention Among Caregivers of Children Under Five

The average score for knowledge toward malaria prevention was 6.94 (SD ± 1.67). More than half of the caregivers (230; 58.4%, 95% CI: 53.5% and 63.0%) demonstrated an adequate level of knowledge regarding malaria prevention. The level of knowledge towards malaria prevention was higher in peri‐urban (129, 64.5%) compared to the rural (101, 52.1%) areas (*p* = 0.012). Caregivers living in the peri‐urban areas, compared to their counterparts in the rural setting, had better knowledge concerning malaria transmitted by mosquito bites (*p* = 0.009), avoiding outdoors at night in order to prevent malaria (*p* = 0.013), symptoms of malaria, including fever (*p* = 0.036) and weight loss (*p* = 0.008), seeking help when symptoms appear (*p* < 0.001), preference for help seeking from health facility (*p* < 0.001) and within 24 h of onset of symptoms (*p* = 0.009). Peri‐urban caregivers knew RDT as a test for malaria (*p* = 0.027) and requesting for it before treatment (*p* = 0.005), and knew the importance of prompt treatment of malaria (*p* < 0.001). Compared to caregivers in peri‐urban areas, those in rural settings showed better knowledge in specific areas related to malaria, such as recognizing the use of mosquito coils as a preventive measure (*p* = 0.002), identifying blood in urine as a symptom of malaria (*p* = 0.029), and preference for seeking care from CHWs (*p* = 0.004) (Table 3).

**TABLE 3:** Knowledge towards malaria prevention among caregivers of children under five in the Buea Health District of Cameroon.

Variable	Category	Peri‐urban	Rural	Total	*p* value
		No (%)	No (%)		
Malaria transmission	Mosquito bite	197 (98.5)	181 (93.3)	378	0.009
	Others	3 (1.5)	13 (6.7)	16	

Methods to avoid getting infected with malaria	Use a mosquito net	183 (91.5)	179 (92.3)	362	0.780
	Avoid being outdoors at night time	33 (16.5)	16 (8.2)	49	0.013
	Apply mosquito repellent	28 (14.0)	25 (12.9)	53	0.746
	Burn mosquito coil	10 (5.0)	27 (13.9)	37	0.002
	Wearing long protective cloth	51 (25.5)	60 (30.9)	111	0.231
	Closing of windows during sunset	21 (10.5)	13 (6.7)	34	0.179

Symptoms of malaria	Fever	189 (94.5)	172 (88.7)	361	0.036
	Headache	116 (58.0)	97 (50.0)	213	0.111
	Body aches	68 (34.0)	78 (40.2)	146	0.202
	Blood in urine	5 (2.5)	14 (7.2)	19	0.029
	Nausea/vomiting	32 (16.0)	29 (14.9)	61	0.773
	Weight loss	14 (7.0)	3 (1.5)	17	0.008
	Loss of appetite	32 (16.0)	22 (11.3)	54	0.179

Seek help when any symptoms are present	Yes	198 (99.0)	167 (86.1)	365	< 0.001
	No	2 (1.0)	27 (13.9)	29	

Preferred biomedical health‐seeking contact	Community health worker	13 (6.5)	30 (15.5)	43	0.004
	Health worker/health facility	161 (80.5)	124 (63.9)	285	< 0.001

Time taken to seek help after signs and symptoms develop	Within 24 h	132 (66.0)	103 (53.1)	235	0.009
	After 24 h	68 (34.0)	91 (46.9)	159	

Heard about severe malaria	Yes	166 (83.0)	167 (86.1)	333	0.398
	No	34 (17.0)	27 (13.9)	61	

Symptoms of severe malaria	Unable to drink	16 (8.0)	14 (7.6)	30	0.769
	Repeated vomiting	43 (21.5)	32 (16.5)	75	0.206
	Anaemia (blood loss)	23 (11.5)	19 (9.8)	42	0.583
	Drowsiness	27 (13.5)	13 (6.7)	40	0.025
	Jaundice	58 (29.0)	7 (3.6)	65	< 0.001
	Convulsions	85 (42.5)	97 (50.0)	182	0.135
	Unconscious	40 (20.0)	58 (29.9)	98	0.023
	Passing no urine	0 (0.0)	2 (1.0)	2	0.150
	Weak or rapid pulse	3 (1.5)	6 (3.1)	9	0.290
	Severe dehydration	2 (1.0)	3 (1.5)	5	0.628
	Bleeding	4 (2.0)	1 (0.5)	5	0.188
	Difficulty in breathing,	11 (5.5)	7 (3.6)	18	0.369
	Neck stiffness	5 (2.5)	4 (2.1)	9	0.771

Knows RDT as test to detect malaria	Yes	24 (12.0)	11 (5.7)	35	0.027
	No	176 (88.0)	183 (94.3)	359	

Request for an RDT before treatment for malaria	Yes	171 (85.5)	144 (74.2)	315	0.005
	No	29 (14.5)	50 (25.8)	79	

Know Coartem as drug to treat malaria	Yes	129 (64.5)	133 (68.6)	262	0.394
	No	71 (35.5)	61 (31.4)	132	

Knows importance of prompt treatment of malaria	Yes	198 (99.0)	173 (89.2)	371	< 0.001
	No	2 (1.0)	21 (10.8)	23	

Knowledge category	Inadequate	71 (35.5)	93 (47.9)	164	0.012
	Adequate	129 (64.5)	101 (52.1)	230	

### 3.4. Attitudes Towards Malaria Prevention Among Caregivers of Children Under Five

The mean score for attitudes towards malaria prevention was 7.10 (SD ± 1.53). Less than half of the caregivers (169; 42.9%, 95% CI: 38.0%, 47.8%) demonstrated positive attitudes towards malaria prevention. The level of positive attitudes towards malaria prevention was higher in peri‐urban (97, 48.5%) compared to the rural (72, 37.1%) areas (*p* = 0.022). Caregivers residing in peri‐urban areas, compared to those in rural settings, were more likely to view malaria as a very serious health problem (*p* = 0.011), emphasize the importance of consistent mosquito bed net use (*p* < 0.001), consider malaria a personal concern (*p* = 0.037), recognize that malaria transmission mainly occurs at night (*p* < 0.001), and value taking a diagnostic test before initiating treatment (*p* = 0.001) (Table 4).

**TABLE 4:** Attitudes towards malaria prevention among caregivers of children under five in the Buea Health District of Cameroon.

Variable	Category	Peri‐urban	Rural	Total	*p* value
		No (%)	No (%)		
Malaria is a very serious health problem	Agree	195 (97.5)	178 (91.8)	373	0.011
	Disagree	5 (2.5)	16 (8.2)	21	

Important to own and always sleeping under a mosquito net	Agree	185 (92.5)	156 (80.4)	341	< 0.001
	Disagree	15 (7.5)	38 (19.6)	53	

Necessary to use treated mosquito net to prevent malaria	Agree	191 (95.5)	178 (91.8)	369	0.127
	Disagree	9 (4.5)	16 (8.2)	25	

Household is affected by malaria	Agree	75 (37.5)	90 (46.4)	165	0.074
	Disagree	125 (62.5)	104 (53.6)	229	

Malaria is an issue of concern for me	Agree	194 (97.0)	179 (92.3)	373	0.037
	Disagree	6 (3.0)	15 (7.7)	21	

Malaria has long‐term consequences on health	Agree	180 (90.0)	165 (85.1)	345	0.137
	Disagree	20 (10.0)	29 (14.9)	49	

Mosquito that transmits malaria bites mostly at night time	Agree	111 (55.5)	72 (37.1)	183	< 0.001
	Disagree	89 (44.5)	122 (62.9)	211	

Important to test for malaria before treatment	Agree	193 (96.5)	169 (87.1)	362	0.001
	Disagree	7 (3.5)	25 (12.9)	32	

Coartem is the best cure for malaria	Agree	143 (71.5)	142 (73.2)	285	0.707
	Disagree	57 (28.5)	52 (26.8)	109	

Attitudes category	Inadequate	103 (51.5)	122 (62.9)	225	0.022
	Adequate	97 (48.5)	72 (37.1)	169	

### 3.5. Malaria Prevention Practices Among Caregivers of Children Under Five

The mean score for practices concerning malaria prevention was 5.16 (SD ± 1.55). Just over half of the caregivers (213; 54.1%, 95% CI: 49.2%–59.0%) showed good practices concerning malaria prevention. The level of good practices about malaria prevention was higher in peri‐urban areas (120, 60.0%) compared to the rural (93, 47.9%) areas (*p* = 0.016). Caregivers living in the peri‐urban areas was reported to be better compared to their counterparts in the rural setting, such as having malaria‐protective methods at home (*p* < 0.001), using window netting (*p* = 0.001), avoiding staying outside during night‐time (*p* = 0.002), using mosquito repellents (*p* = 0.001), requesting a malaria test before treating their child (*p* < 0.001), and adhering to the prescribed duration of treatment for children diagnosed with malaria (*p* < 0.001). Compared to caregivers in peri‐urban areas, those in rural settings exhibited better practices in certain aspects of malaria prevention, including clearing bushes around the house (*p* < 0.001) and seeking care from a health worker when their child showed symptoms of malaria (*p* = 0.002) (Table 5).

**TABLE 5:** Malaria prevention practices among caregivers of children under five in the Buea Health District of Cameroon.

Variable	Category	Peri‐urban	Rural	Total	*p* value
		No (%)	No (%)		
Household with malaria protective method	Yes	194 (97.0)	157 (80.9)	351	< 0.001
	No	6 (3.0)	37 (19.1)	43	

Protective method against malaria	Sleep under a mosquito bed net	151 (75.5)	134 (69.1)	285	0.154
	Use netting on windows	28 (14.0)	8 (4.1)	46	0.001
	Avoid outside during night‐time	24 (12.0)	7 (3.6)	31	0.002
	Use insecticide spray	48 (24.0)	32 (16.5)	80	0.064
	Wear long protective clothing	50 (25.0)	61 (31.4)	111	0.155
	Use mosquito repellent	33 (16.5)	12 (6.2)	45	0.001
	Clearing bushes around the house	43 (21.5)	79 (40.7)	122	< 0.001
	Getting rid of stagnant water	51 (25.5)	38 (19.6)	89	0.161
	Regular clean‐ups around the house	44 (22.0)	45 (23.2)	89	0.777

Claims to always use mosquito bed nets	Yes	135 (67.5)	123 (63.4)	258	0.392
	No	65 (32.5)	71 (36.6)	136	

Under‐five children slept under a mosquito net the previous night	Yes	108 (54.0)	101 (52.1)	209	0.700
	No	92 (46.0)	93 (47.9)	185	

Take child to health worker when symptoms of malaria are present	Yes	58 (29.0)	86 (44.3)	144	0.002
	No	142 (71.0)	108 (55.7)	250	

Request for malaria test before treating my child	Yes	178 (89.0)	139 (71.6)	317	< 0.001
	No	22 (11.0)	55 (28.4)	77	

Give medication to child as prescribed by the health professional	Yes	195 (97.5)	186 (95.9)	381	0.367
	No	5 (2.5)	8 (4.1)	13	

Respect number of days prescribed by health professional	Yes	185 (92.5)	154 (79.4)	339	< 0.001
	No	15 (7.5)	40 (20.6)	55	

Practices	Poor	80 (40.0)	101 (52.1)	181	0.016
	Good	120 (60.0)	93 (47.9)	213	

### 3.6. Factors Associated With Adequate Knowledge of Malaria Prevention Among Caregivers of Children Under Five in the BHD of Cameroon

Table 6 presents the results of the fitted bivariate and multivariate logistic model using enter method to control for age, sex, respondent relationship with the child, marital status, level of education, income, household assets, religion, and number of persons per household.

**TABLE 6:** Factors associated with adequate knowledge on malaria prevention among the caregivers of children under five in the Buea Health District of Cameroon.

Variable	Category	Level of knowledge		COR	95% CI	*p* value	AOR	95% CI	*p* value
		Inadequate	Adequate						
		No (%)	No (%)						
Sex of respondent	Female	140 (41.7)	196 (58.3)	1.00	—	—	1.00		
	Male	24 (41.4)	34 (58.6)	1.01	0.68–1.78	0.967			
Respondent’s relationship with the child	Caregiver/head of household	139 (39.8)	210 (60.2)	1.43	0.57–3.56	0.444	2.23	0.82–6.11	0.118
	Caregiver	12 (44.4)	15 (55.6)	1.47	0.77–2.79	0.046	2.23	1.10–4.49	0.026
	Head of household	10 (76.9)	3 (23.1)	1.00	—	—	1.00	—	—
Age of father (years)	< 21	2 (100.0)	0 (0.0)		—	—			
	21–30	35 (56.5)	27 (43.5)	0.68	0.35–1.33	0.258			
	31–40	65 (32.7)	134 (67.3)	1.81	1.08–3.09	0.028			
	> 40	37 (46.8)	42 (53.2)	1.00	—	—	1.00		
Age of mother (years)	< 21	12 (63.2)	7 (36.8)	0.75	0.20–2.92	0.678			
	21–30	79 (45.7)	94 (54.3)	1.53	0.55–4.29	0.419			
	31–40	58 (33.5)	115 (66.5)	2.55	0.90–7.19	0.077			
	> 40	9 (56.3)	7 (43.8)	1.00	—	—	1.00		
Religion of household	Muslim	9 (75.0)	3 (25.0)	1.00	—	—	1.00		
	None	0 (0.0)	10 (100.0)	—	—	—	—	—	—
	Pentecostal	55 (45.8)	65 (54.2)	3.54	0.91–13.75	0.067	4.02	1.00–16.12	0.050
	Protestant	72 (41.1)	103 (58.9)	4.29	1.12–16.41	0.033	5.45	1.38–21.62	0.016
	Roman Catholic	28 (36.4)	49 (63.6)	5.25	1.31–21.01	0.019	6.53	1.57–27.31	0.010
Relationship of respondent with the under fives in the household	Brother/sister’s child	12 (80.0)	3 (20.0)	0.17	0.05–0.60	0.006	0.16	0.04–0.61	0.007
	Grandchild	15 (55.6)	12 (44.4)	0.83	0.37–1.82	0.638	0.75	0.32–1.73	0.505
	Son‐in‐law/daughter‐in‐law’s child	1 (33.1)	1 (66.7)	1.32	0.12–14.74	0.821	1.17	0.10–14.20	0.905
	Son/daughter	173 (49.6)	176 (50.4)	1.00	—	—	1.00	—	—
Highest completed level of education of father	None	4 (66.7)	2 (33.3)	1.00	—	—	1.00		
	Primary	37 (63.8)	21 (36.2)	1.14	0.19–6.73	0.889			
	Secondary	95 (37.8)	156 (62.2)	3.28	0.59–18.28	0.175			
	Tertiary	10 (23.8)	32 (76.2)	6.40	1.02–40.29	0.048			
Highest completed level of education of mother	None	4 (80.0)	1 (20.0)	1.00	—	—	1.00	—	—
	Primary	54 (64,3)	30 (35.7)	2.22	0.24–20.80	0.484			
	Secondary	92 (36.2)	162 (63.8)	7.04	0.78–63.96	0.083			
	Tertiary	11 (26.2)	31 (73.8)	11.27	1.13–112.07	0.039			
Main occupation	Employed	13 (26.5)	36 (73.5)	10.15	2.44–42.25	0.001	8.12	1.86–35.28	0.005
	Housewife	6 (60.0)	4 (40.0)	2.44	0.40–14.75	0.330	1.88	0.28–12.29	0.509
	Self‐employed	129 (41.9)	179 (58.1)	5.09	1.39–18.60	0.014	4.88	1.30–18.38	0.019
	Student	5 (38.5))	8 (61.7)	5.88	1.08–32.00	0.041	7.66	1.27–46.12	0.026
	Unemployed	11 (78.6)	3 (21.0)	1.00	—	—	1.00	—	—
Household’s estimated monthly income (FCFA)	< 50,000	119 (42.2)	163 (57.8)	1.00	—	—	1.00	—	—
	50–100,000	35 (37.6)	58 (62,4)	1.21	0.78–1.96	0.438			
	> 100,000	10 (52.6)	9 (47.4)	0.66	0.26–1.67	0.377			
Number of persons living in household	1–3	27 (50.9)	26 (49.1)	1.00	—	—			
	4–6	98 (39.5)	150 (60.5)	1.59	0.88–2.88	0.127			
	6–8	25 (41.0)	36 (59.0)	1.50	0.71–3.14	0.288			
	> 8	14 (43.8)	18 (56.3)	1.34	0.55–3.23	0.521			
Type of asset owned by household	Mobile phone	156 (41.2)	223 (58.8)	1.63	0.58–4.59	0.353			
	Radio	43 (39.4)	59 (57.8)	1.03	0.65–1.63	0.899			
	Television	135 (39.4)	208 (60.6)	2.03	1.12–3.68	0.020	1.58	0.85–2.94	0.147
Type of material used for house construction	Brick	52 (32.1)	110 (67.9)	1.974	1.30–3.00	0.001	1.88	1.20–2.95	0.006
	Plank	112 (48.3)	120 (51.7)	1.00	—	—	1.00	—	—
Type of floor in the house	Cement	143 (44.4)	179 (55.6)	1.00	—	—			
	Tiles	9 (60.0)	6 (40.0)	0.533	0.19–1.53	0.242	0.71	0.23–2.14	0.539
	Mud	12 (21.1)	45 (78.9)	2.996	1.53–5.88	0.001	2.62	1.29–5.32	0.008

Caregivers were two times more knowledgeable about malaria prevention in under‐five children compared to heads of households [AOR 2.23, 95% CI 1.10–4.49, *p* = 0.026]. The odds of having adequate knowledge on malaria prevention was about six (6 times) higher among caregivers of children under five who Christians (Roman Catholics [AOR 6.53, 95% CI 1.57–27.31, *p* = 0.010] and Protestants [AOR 6.53, 95% CI 1.38–21.62, *p* = 0.016]) by religion compared to caregivers who were Muslims. Caregivers who were employed [AOR 8.12, 95% CI 1.86–35.28, *p* < 0.01] were eight times more knowledgeable on malaria prevention in children under five compared to those who were unemployed. In comparison to type of material used for house construction, caregivers in houses made of bricks [AOR 1.88, 95% CI 1.20–2.95, *p* < 0.01] were more knowledgeable about malaria prevention in children under five compared to those in plank houses.

### 3.7. Factors Associated With Positive Attitudes Towards Malaria Prevention Among Caregivers of Children Under Five in the BHD of Cameroon

Caregivers of children under five who were employed [AOR 8.27, 95% CI 1.49–45.97, *p* = 0.016] eight times more likely to have positive attitudes on malaria prevention compared to those who were unemployed. The odds of having positive attitudes on malaria prevention was five times higher in caregivers who had mobile phones [AOR 5.71, 95% CI 1.24–26.20, *p* = 0.025] as household asset compared to those who did not. Compared to cement floor type, caregivers of children under five with tiles [AOR 1.88, 95% CI 1.05–3.36, *p* = 0.035] as floor types were more likely to have positive attitudes on malaria prevention (Table 7).

**TABLE 7:** Factors associated with positive attitudes towards malaria prevention among the caregivers of children under five in the Buea Health District of Cameroon.

Variable	Category	Level of attitudes		COR	95% CI	*p* value	AOR	95% CI	*p* value
		Negative	Positive						
		No (%)	No (%)						
Sex of respondent	Female	193 (57.4)	143 (42.6)	1.00	—				
	Male	32 (55.2)	26 (44.8)	1.10	0.63–1.92	0.750			
Respondent’s relationship with the child	Caregiver/head of household	15 (44.1)	19 (55.9)	1.86	0.74–4.65	0.183			
	Caregiver	185 (58.2)	133 (41.8)	1.05	0.54–2.03	0.868			
	Head of household	25 (59.5)	17 (40.5)	1.00	—	—			
Age of father (years)	< 21	1 (50.0)	1 (50.0)	1.32	0.08–21.92	0.845			
	21–30	32 (51.6)	30 (48.4)	1.24	0.63–2.42	0.527			
	31–40	116 (58.3)	83 (41.7)	0.95	0.55–1.60	0.840			
	> 40	45 (57.0)	34 (43.0)	1.00	—	—			
Age of mother (years)	< 21	13 (68.4)	6 (31.6)	0.46	0.12–1.82	0.271			
	21–30	97 (56.1)	76 (43.9)	0.78	0.28–2.18	0.641			
	31–40	100 (57.8)	73 (42.2)	0.73	0.26–2.03	0.547			
	> 40	8 (50.0)	8 (50.0)	1.00	—	—			
Religion of household	Muslim	6 (50.0)	6 (50.0)	1.00	—	—			
	None	7 (70.0)	3 (30.0)	0.43	0.07–2.50	0.346			
	Pentecostal	62 (51.7)	58 (48.3)	0.94	0.29–3.06	0.912			
	Protestant	102 (58.3)	73 (41.7)	0.72	0.22–2.30	0.575			
	Roman Catholic	48 (62.3)	29 (37.7)	0.60	0.18–2.05	0.419			
Relationship of respondent with the under fives in the household	Son/daughter	206 (58.9)	144 (41.1)	1.00	—	—			
	Grandchild	10 (35.7)	18 (64.3)	0.71	0.23–2.13	0.543	0.13	0.03–0.61	0.010
	Brother/sister’s child	9 (69.2)	4 (30.8)	2.85	1.24–6.52	0.013	0.83	0.35–1.96	0.667
	Son‐in‐law/daughter‐in‐law’s child	0 (0.0)	2 (100.0)	2.85	0.25–31.69	0.395	0.33	0.03–4.14	0.388
Highest completed level of education of father	None	4 (66.7)	2 (33.3)	1.00	—	—			
	Primary	29 (50.0)	29 (50.0)	2.00	0.34–11.79	0.444			
	Secondary	149 (59.4)	102 (40.6)	1.37	0.25–7.62	0.720			
	Tertiary	21 (50.0)	21 (50.0)	2.00	0.33–12.12	0.451			
Highest completed level of education of mother	None	3 (60.0)	2 (40.0)	1.00	—	—			
	Primary	48 (57.1)	36 (42.9)	1.13	0.18–7.08	0.900			
	Secondary	146 (57.5)	108 (43.5)	1.11	0.18–6.76	0.910			
	Tertiary	22 (52.4)	20 (47.6)	1.37	0.21–9.02	0.748			
Main occupation	Employed	21 (59.2)	20 (40.8)	2.53	0.62–10.23	0.193	8.27	1.49–45.97	0.016
	Housewife	6 (60.0)	4 (40.0)	2.44	0.40–14.74	0.330	5.92	0.73–48.19	0.096
	Self‐employed	170 (55.2)	138 (44.8)	2.97	0.81–10.88	0.049	5.86	1.22–28.24	0.028
	Student	9 (69.2)	4 (30.8)	1.63	0.28–9.25	0.582	5.65	0.79–40.54	0.085
	Unemployed	11 (73.6)	3 (21.4)	1.00	—	—			
Household’s estimated monthly income (FCFA)	< 50,000	169 (59.9)	113 (40.1)	1.00	—	—			
	50–100,000	48 (51.6)	45 (48.4)	1.42	0.88–2.25	0.160			
	> 100,000	8 (42.1)	11 (57.9)	2.06	0.80–5.27	0.133			
Number of persons living in household	1–3	31 (58.5)	22 (41.5)	1.00	—	—			
	4–6	148 (59.7)	100 (40.3)	0.95	0.52–1.74	0.873			
	6–8	30 (49.2)	31 (50.8)	1.46	0.69–3.06	0.321			
	> 8	16 (50.0)	16 (50.0)	1.41	0.58–3.41	0.446			
Type of asset owned by household	Radio	55 (53.9)	47 (46.1)	1.19	0.76–1.87	0.451			
	Television	212 (55.9)	167 (44.1)	0.63	0.35–1.13	0.122			
	Mobile phone	201 (58.6)	142 (41.4)	5.12	1.14–23.00	0.033	5.71	1.24–26.20	0.025
Type of material used for house construction	Brick	92 (56.8)	70 (43.2)	1.02	0.68–1.53	0.916			
	Plank	133 (57.3)	99 (42.7)	1.00	—	—			
Type of floor in the house	Cement	192 (59.6)	130 (40.4)	1.00	—	—			
	Mud	25 (43.9)	32 (56.1)	1.29	0.46–3.65	0.628	1.41	0.49–4.02	0.524
	Tiles	8 (53.3)	7 (46.7)	1.89	1.071–3.33	0.028	1.88	1.05–3.36	0.035

### 3.8. Factors Associated With Good Practices About Malaria Prevention Among Caregivers of Children Under Five in the BHD of Cameroon

Caregivers of children under five who were employed [AOR 6.97, 95% CI 1.26–38.51, *p* = 0.025] were six times more likely to have good practices on malaria prevention compared to those who were unemployed. The odds of having good practices on malaria prevention was about five times higher in caregivers who had mobile phones [AOR 4.79, 95% CI 1.06–21.68, *p* = 0.042] as household assets compared to those who did not. In comparison to type of material used for house construction, caregivers in houses made of bricks [AOR 1.88, 95% CI 1.20–2.95, *p* < 0.01] were two times more likely to have good practices on malaria prevention in children under five than those in plank houses (Table 8).

**TABLE 8:** Factors associated with good practices about malaria prevention among caregivers of children under five in the Buea Health District of Cameroon.

Variable	Category	Level of practices		COR	95% CI	*p* value	AOR	95% CI	*p* value
		Poor	Good						
		No (%)	No (%)						
Sex of respondent	Female	150 (44.6)	186 (55.4)	1.00	—	—			
	Male	31 (53.4)	27 (46.6)	0.70	0.40–1.23	0.215			

Respondent’s relationship with the child	Caregiver/head of household	21 (61.8)	13 (38.2)	0.68	0.27–1.71	0.413			
	Caregiver	138 (43.4)	18 (56.6)	1.44	0.75–2.73	0.273			
	Head of household	22 (52.4)	20 (47.6)	1.00	—	—			

Age of father (years)	< 21	2 (100.0)	0 (0.0)	—	—	—			
	21–30	31 (50.0)	31 (50.0)	0.65	0.33–1.27	0.202			
	31–40	81 (40.7)	118 (59.3)	0.94	0.55–1.60	0.823			
	> 40	31 (39.2)	48 (60.8)	1.00	—	—			

Age of mother (years)	< 21	13 (68.4)	6 (31.6)	0.28	0.07–1.12	0.072			
	21–30	87 (50.3)	86 (49.7)	0.59	0.21–1.70	0.332			
	31–40	65 (37.6)	108 (62.4)	0.99	0.35–2.87	0.995			
	> 40	6 (37.5)	10 (62.5)	1.00	—	—			

Religion of household	Muslim	4 (33.3)	8 (66.7)	1.00	—	—			
	None	6 (60.0)	4 (40.0)	0.33	0.06–1.91	0.217			
	Pentecostal	65 (54.2)	55 (45.8)	0.42	0.12–1.48	0.178			
	Protestant	73 (41.7)	102 (58.3)	0.70	0.20–2.40	0.570			
	Roman Catholic	33 (42.9)	44 (57.1)	0.67	0.19–2.40	0.535			

Relationship of respondent with the under fives in the household	Son/daughter	153 (43.7)	197 (56.3)	1.00	—	—			
	Grandchild	13 (86.7)	21 (1332)	6.04	1.14–32.04	0.035	0.71	0.23–2.17	0.546
	Brother/sister’s child	14 (51.9)	13 (48.1)	3.25	0.19–54.78	0.413	2.74	1.19–6.29	0.018
	Son‐in‐law/daughter‐in‐law’s child	2 (66.7)	1 (33.3)	8.42	1.87–37.89	0.005	2.48	0.22–28.21	0.465

Highest completed level of education of father	None	5 (83.3)	1 (16.7)	1.00	—	—			
	Primary	41 (70.7)	17 (29.3)	2.073	0.22–19.09	0.520			
	Secondary	97 (38.6)	154 (61.4)	7.938	0.91–68.97	0.060			
	Tertiary	16 (38.1)	28 (61.9)	8.125	0.87–75.98	0.066			

Highest completed level of education of mother	None	4 (80.0)	1 (20.0)	1.00	—	—			
	Primary	55 (65.5)	29 (34.5)	2.12	0.23–19.75	0.513			
	Secondary	102 (40.2)	152 (59.8)	5.96	0.66–54.09	0.113			
	Tertiary	16 (38.1)	26 (61.9)	6.50	0.67–63.42	0.107			

Main occupation	Employed	12 (41.4)	17 (58.6)	10.33	2.07–51.47	0.004	6.97	1.26–38.51	0.026
	Housewife	6 (33.3)	12 (66.7)	6.00	0.86–41.90	0.071	9.01	0.98–82.47	0.052
	Self‐employed	50 (50.5)	49 (49.5)	7.295	1.61–33.14	0.010	6.04	1.28–28.48	0.023
	Student	88 (42.5)	119 (57.5)	5.143	0.81–32.77	0.083	6.53	0.83–51.05	0.074
	Unemployed	25 (61.0)	16 (39.0)	1.00	—	—			

Household’s estimated monthly income (FCFA)	< 50,000	134 (47.5)	148 (52.5)	1.00	—	—			
	50–100,000	39 (41.9)	54 (58.1)	1.25	0.78–2.01	0.349			
	> 100,000	8 (42.1)	11 (57.9)	1.25	0.49–3.19	0.648			

Number of persons living in household	1–3	26 (49.1)	27 (50.9)	1.00	—	—			
	4–6	100 (40.3)	148 (59.7)	1.43	0.79–2.59	0.243			
	6–8	33 (54.1)	28 (45.9)	0.82	0.39–1.71	0.591			
	> 8	22 (68.8)	10 (31.3)	0.44	0.17–1.10	0.079			

Type of asset owned by household	Radio	42 (41.2)	60 (58.8)	1.30	0.82–2.05	0.263			
	Television	148 (43.1)	195 (56.9)	2.42	1.31–4.46	0.005			
	Mobile phone	173 (45.6)	206 (54.4)	1.361	0.48–3.83	0.559	4.79	1.06–21.68	0.042

Type of material used for house construction	Brick	59 (36.4)	103 (63.6)	1.94	1.28–2.92	0.002	2.21	1.36–3.61	0.002
	Plank	122 (52.6)	110 (47.4)	1.00	—	—			

Type of floor in the house	Cement	153 (47.5)	169 (52.5)	1.00	—	—			
	Mud	19 (60.0)	38 (40.0)	0.60	0.21–1.74	0.349	1.32	0.46–3.83	0.611
	Tiles	19 (33.3)	38 (66.7)	1.81	1.00–3.28	0.050	1.87	1.05–3.34	0.034

## 4. Discussion

This study assessed the KAP of caregivers of children under‐five years towards malaria prevention and identified factors associated with adequate knowledge, positive attitudes, and good practices in the BHD. The results of this study showed that a little over half of the caregivers demonstrated adequate knowledge about malaria prevention, a proportion similar to that reported in other rural areas of the South West region of Cameroon [11] and slightly higher than that observed among internally displaced persons and returnee populations in conflict‐affected communities [6]. However, the proportion for adequate knowledge in the current study is higher than that reported among communities in the Western administrative Region of Cameroon of comparable altitudes [7] and substantially higher than that observed in Nigerian and Ugandan settings [12, 13]. The relatively higher levels in Buea reflect better health service functionality, higher literacy rates, and greater access to mass media in peri‐urban areas compared to conflict or hard‐to‐reach settings.

In this study, adequate knowledge was associated with being a caregiver rather than the household head, being employed, and living in better conditions. Similar trends have been observed in other Cameroonian contexts where caregivers directly responsible for child care tend to have greater awareness of preventive measures [6, 11]. Employment has been linked to improved knowledge through its role in enhancing access to health information and resources [6, 14] while living in better conditions such as brick houses reduces mosquito entry and is often a proxy for better socioeconomic status, which has also been reported as an enabling factor in Nigeria [12]. Our findings suggest, however, that it is not the type of house in itself that determines caregivers’ KAP, but rather the socioeconomic status that such housing reflects. Caregivers living in poorer housing conditions were more likely to be economically disadvantaged, which limited affordability of preventive tools and was often associated with lower levels of formal education which is a key mediator of knowledge. Likewise, ownership of assets such as a television is better understood as an indicator of socioeconomic well‐being and media exposure rather than a direct causal factor. These patterns reinforce the interpretation that housing quality and asset ownership co‐occur with broader socioeconomic advantage, rather than acting as independent determinants of malaria‐related KAP.

Positive attitudes were more likely among caregivers in peri‐urban areas, those with secondary or tertiary education, and those owning a television set (this is just an indicator of better living conditions which is neither a factor nor a proxy). The peri‐urban effect echoes findings from other KAP studies in Cameroon [7, 11, 14], where greater exposure to health campaigns and easier access to health services improve risk perception. Education has repeatedly been linked to stronger preventive attitudes [7, 13], likely due to improved understanding of disease causation and benefits of control measures. Television ownership in itself can be a predictor if associated with health campaigns or exposure to health education and would be consistent with the role of mass media in shaping health attitudes reported in SSA [9].

A little over half of caregivers reported good preventive practices, which is a good threshold level for acceptable practices. Here, close to half do not report good practices, which is not a good indicator of progress. This figure is higher than what has been documented in southern Cameroon forest communities [14] and in conflict‐affected areas of Cameroon [11], but similar disparities between peri‐urban and rural households have been noted elsewhere [6, 14]. Rural households often face structural constraints, such as poor housing, inadequate mosquito bed net coverage, and limited replacement cycles, which undermine consistent practice even where knowledge is present [12, 14]. In our study, good practices were more likely among peri‐urban caregivers, those with adequate knowledge and positive attitudes, mothers with at least secondary education, and those who reported requesting malaria testing before treatment. The association between good practices and testing aligns with WHO guidelines promoting universal parasitological confirmation [15] and with Ugandan evidence linking testing to better treatment adherence [13].

Overall, the findings reinforce the interconnectedness of KAP while highlighting the fact that socioeconomic and environmental factors shape malaria prevention behaviour. As seen in other Cameroonian studies [7, 11, 14], sustainable improvement will require interventions that go beyond traditional awareness campaigns to address attitudinal barriers and reduce inequities in access to prevention tools. Given the high level of mobile phone ownership observed among caregivers (96.2%), mobile health (mHealth) interventions may represent a potentially feasible approach for delivering malaria prevention messages. However, this study did not assess caregivers’ engagement with mobile‐based interventions or their impact on behaviour. As such, further research is needed to evaluate the acceptability, feasibility, and effectiveness of SMS‐based health education in this context. Similar mobile‐based interventions in other low‐resource settings have been shown to improve maternal and child health behaviours [16–18], suggesting this approach could be adapted to strengthen malaria prevention among caregivers in the BHD.

A key strength of this study is its community‐based design, which involved peri‐urban and rural communities of the BHD. This allowed for a direct comparison of KAP disparities across different residential settings. The relatively large sample size enhanced the statistical power to detect associations, while the use of a pre‐tested structured questionnaire improved the reliability and consistency of data collection. Furthermore, the inclusion of multiple sociodemographic, socioeconomic, and environmental variables provided a more holistic understanding of the determinants of malaria KAP. However, the study has limitations. The cross‐sectional design limits the ability to infer causality between the identified factors and KAP outcomes. The reliance on self‐reported practices may have introduced social desirability bias, potentially overestimating the true proportion of good practices. Seasonal variation in malaria transmission was not controlled for, which may have influenced both actual practices and perceived risk. Finally, while the findings are relevant for the BHD, generalization to other parts of Cameroon can only be made with caution, particularly in regions with different sociopolitical and epidemiological profiles.

## 5. Conclusion

This study revealed that while over half of caregivers possess adequate knowledge of malaria prevention, notable gaps persist in attitudes and practices, especially among rural residents in the BHD. These gaps are shaped by socioeconomic, educational, and structural factors, highlighting the need for interventions that go beyond traditional awareness campaigns. Given the near‐universal ownership of mobile phones among caregivers, mHealth represents a potentially promising platform for delivering malaria prevention messages. However, further studies are needed to assess its feasibility, acceptability, and effectiveness in improving caregiver KAP. Delivering structured, context‐specific malaria prevention messages via text messaging can provide caregivers with timely reminders, reinforce key prevention behaviours and address misconceptions. This approach has the potential to strengthen knowledge, foster positive attitudes, and sustain preventive practices across both peri‐urban and rural settings overcoming barriers related to distance from health facilities and limited access to health workers.

## Author Contributions

M.N.L., E.Y.V., and D.S.N.: conceptualization; M.N.L., E.A.T., E.Y.V., and D.S.N.: methodology; writing–original draft preparation; M.N.L., E.A.T., E.Y.V., and D.S.N.: investigation; M.N.L. and T.E.A.: data analysis; M.N.L., E.A.T., E.Y.V., and D.S.N.: writing–review and editing; E.Y.V. and D.S.N.: supervision and project administration.

## Funding

No specific funding was received for this study.

## Disclosure

All authors have read and agreed to the published version of the manuscript. This is part of an unpublished PhD thesis conducted on “Text messaging health education intervention on malaria control among caregivers of children under five in the Buea Health District: A cluster‐randomized controlled trial” at the Faculty of Health Sciences, University of Buea in Cameroon [19].

## Ethics Statement

The protocol was approved by the Institutional Review Board bearing number 2024/1975‐02/UB/SG/IRB/FHS.

## Consent

Informed consent was obtained from all participants prior to the start of data collection.

## Conflicts of Interest

The authors declare no conflicts of interest.

## Supporting Information

Additional supporting information can be found online in the Supporting Information section.

## Supporting information


**Supporting Information 1** Structured questionnaire used to assess knowledge, attitudes, and practices of caregivers towards malaria prevention.

## Data Availability

The data that support the findings of this study are available from the corresponding author upon reasonable request.
