# Supplementary material for: A Formative Cross‐Sectional Study to Assess the Knowledge, Attitude, and Practices of Caregivers of Children Under Five Towards Malaria Control in the Buea Health District of Cameroon
Source: J Trop Med. 2026 Jun 30;2026:5566468. doi: 10.1155/jotm/5566468 (PMC13318479; doi:10.1155/jotm/5566468)
Supplement: Supplementary file 1 — Supporting Information 1 Structured questionnaire used to assess knowledge, attitudes, and practices of caregivers towards malaria prevention. [file JOTM-2026-5566468-s001.docx]

**QUESTIONNAIRE for the study: A formative cross-sectional study to assess the knowledge, attitude and practices of caregivers of children under-five towards malaria control in the Buea Health District of Cameroon**

**Section A: Socio-demographic characteristics of caregiver**

| **No** | **Question** | **Response (Enter the number corresponding to the**  **correct response in the check box)** | **Remark** |
| --- | --- | --- | --- |
| Q1 | Age of father (years) | [ \| ] NA [__] |  |
| Q2 | Age of mother (years) | [ \| ] NA [__] |  |
| Q3 | Sex of head of household | [__]  1= Male 2= Female |  |
| Q4 | **What is your religion?**  SINGLE RESPONSE | None [ ]  Christian (Protestant) [ ]  Christian (Roman Catholic) [ ]  Christian (Pentecostal) [ ]  Muslim [ ]  Other Specify______________ |  |
| Q5 | **What is your relationship with the under-fives in this household?**  MULTIPLE RESPONSE IN CASE OF MORE THAN ONE CHILD | Son/daughter [ ]  Adopted/foster child [ ]  Son-in-law/daughter-in-law’s child [ ]  Grandchild [ ]  Brother/sister’s child [ ]  Other Specify ______________________ |  |
| Q6 | **What is the highest level of education the father has completed?**  SINGLE RESPONSE | None [ ]  Primary [ ]  Secondary [ ]  Tertiary [ ]  Other Specify: _______________________ |  |
| Q7 | **What is the highest level of education the mother has completed?**  SINGLE RESPONSE | None [ ]  Primary [ ]  Secondary [ ]  Tertiary [ ]  Other Specify: _____________________ |  |
| Q8 | **What is your main occupation?**  SINGLE RESPONSE | None [ ]  Public sector employee [ ]  Private sector employee [ ]  Self-employed (business) [ ]  Self-employed (agriculture/farming) [ ]  Teacher [ ]  Student [ ]  Other Specify: ______________________ |  |
| Q9 | **Estimated monthly income (FCFA)** | [ \| \|__\| \| \|__\| ] |  |
| Q10 | **Number of persons living in household** | [ \| ] |  |
| Q11 | **Number of under-five years children living in household** | [ \| ] |  |
| Q12 | **Does your household have any of the following assets?**  ASK ABOUT EACH OF THE ASSESTS AND CIRCLE | Yes No  Radio    Television  Mobile phone |  |
| Q13 | **Type of material used for house construction** | Brick [ ]  Caraboat [ ] |  |
| Q14 | **Type of floor in the house** | Cement [ ]  Tiles [ ]  Mud [ ] |  |
| Q15 | **Type of place of residence** | Rural [ ]  Peri-urban [ ] |  |

**Section B: Socio-demographic characteristics of under-five child(ren)**

|  |  | Sex of child | Age of child (in months)  (Please estimate if unknown) |  |
| --- | --- | --- | --- | --- |
| Q16a | **Child 1** | Male [ ]  Female [ ] | [____\|___] |  |
| Q16b | **Child 2** | Male [ ]  Female [ ] | [____\|___] |  |
| Q16c | **Child 3** | Male [ ]  Female [ ] | [____\|___] |  |
| Q16d | **Child 4** | Male [ ]  Female [ ] | [____\|___] |  |
| Q16e | **Child 5** | Male [ ]  Female [ ] | [____\|___] |  |

**Section C: Caregiver’s Knowledge of Malaria Prevention and care**

| Q22 | **Have you heard of malaria?**  SINGLE RESPONSE | Yes [ ]  No [ ] |  |
| --- | --- | --- | --- |
| Q23 | **In the last six months, where did you hear about it?**  MULTIPLE RESPONSES POSSIBLE  CIRCLE ALL MENTIONED  PROBE TWICE: ANYTHING ELSE? | Not heard about malaria in the last  six months [ ]  Health professional [ ]  Community health worker [ ]  Community meeting [ ]  School [ ]  Radio/TV [ ]  Newspaper [ ]  Relative/friend/neighbour [ ]  Other Specify:_______________________ |  |
| Q24 | **How can someone get infected with malaria?**  MULTIPLE RESPONSES POSSIBLE  PROBE TWICE: ANYTHING ELSE? | Mosquito bite [ ]  Staying under the sun for long [ ]  Eating dirt [ ]  Other Specify:_____________________ |  |
| Q25 | **Do you know how you can avoid getting malaria?**  MULTIPLE RESPONSES POSSIBLE  PROBE ONCE: ANYTHING ELSE? | Use a mosquito net [ ]  Avoid being outdoors at night time [ ]  Apply mosquito repellent [ ]  Burn mosquito coil [ ]  Wearing long protective cloth [ ]  Use of fan [ ]  Closing of windows during sunset [ ]  Burning of traditional herbs [ ]  Other Specify _______________________    Others [ ]  Specify:___________________________ |  |
| Q26 | **What are the possible symptoms of malaria?**  MULTIPLE RESPONSES POSSIBLE  PROBE ONCE: ANYTHING ELSE? | Fever [ ]  Headache [ ]  Body aches [ ]  Blood in urine [ ]  Diarrhoea [ ]  Nausea/vomiting [ ]  Rash/itch [ ]  Weight loss [ ]  Loss of appetite [ ]  Other Specify:____ _________________ |  |
| Q27 | **If you have any of the above symptoms, would you seek help?**  SINGLE RESPONSE | Yes [ ]  No [ ]  Don’t know …………………………...[ ] |  |
| Q28 | **If you were to ask for help, who would you look for help?**  MULTIPLE RESPONSES POSSIBLE  PROBE ONCE: ANYONE ELSE? | Spiritual leader [ ]  Traditional healer [ ]  Pharmacy [ ]  Drug vendor in the quarter [ ]  Community health worker [ ]  Health worker/health facility [ ]  Family member [ ]  Other Specify: ______________________ |  |
| Q29 | **How long should you stay after noticing the signs and symptoms?**  SINGLE RESPONSE | Same day . . . . . . . . . . . . . . . . . . . . . . . [__]  Next day . . . . . . . . . . . . . . . . . . . . . . . . [__]  Two days after fever . . . . . . . . . . . . . . [__] Three or more days after fever . . . . . [__] |  |
| Q30 | **If No to Q28, What are some of the reasons that can make you not to ask for help?**  MULTIPLE RESPONSES POSSIBLE  PROBE ONCE: ANY OTHER REASON? | I don’t have anyone to go to [ ]  I have no money [ ]  I am not concerned about the symptoms [ ]  I never seek medical help [ ]  I can treat the sickness by myself [ ]  Other Specify:______________________ |  |
| Q31 | **Have you heard of severe malaria?** | Yes [ ]  No [ ] |  |
| Q32 | **If Yes to Q31,**  **How will you know if someone has severe malaria?**  MULTIPLE RESPONSES POSSIBLE  PROBE ONCE: ANYONE ELSE? | Unable to drink [ ]  Repeated vomiting [ ]  Anemia (blood loss) [ ]  Drowsiness [ ]  Jaundice [ ]  Purging [ ]  Convulsions [ ]  Unconscious [ ]  Passing no urine [ ]  Weak or rapid pulse [ ]  Coughing [ ]  Severe dehydration [ ]  Bleeding [ ]  Difficulty breathing, [ ]  Neck stiffness [ ]  Others Specify [ ] |  |
| Q33 | **Do you know the test for malaria?**  SINGLE RESPONSE  IF RESPONDENT DOES NOT KNOW THE NAME OF THE DRUG, SAY “THE TEST USED TO TREAT MALARIA IS CALLED AN RDT” | Yes, I know the name [ ]  No, I don’t know the name [ ] |  |
| Q34 | **Should an RDT be conducted before being treated for malaria?** | Yes, [ ]  No [ ] |  |
| Q35 | **Do you know the name of the drug to treat malaria?**  SINGLE RESPONSE  IF RESPONDENT DOES NOT KNOW THE NAME OF THE DRUG, SAY “THE DRUG USED TO TREAT MALARIA IS CALLED COARTEM” | Yes, I know the name [ ]  No, I don’t know the name [ ] |  |
| Q36 | **Do you think taking drugs to treat malaria is always necessary** | Yes [ ]  No [ ] |  |
| Q37 | **If yes to Q36, Why?** | It is necessary to always treat malaria [ ]  To prevent further complications [ ]  To get well [ ]  Others Specify ______________________ |  |
| Q38 | **If No to Q36, Why not?** | The malaria will go on its own [ ]  Just rest and eat well [ ]  Don’t drink alcohol [ ]  Others Specify _______________________ |  |

**Section D: Attitudes towards malaria Prevention**

| Q39 | **Do you think malaria is a very serious health problem?** | Yes [ ]  No [ ]  Not sure [ ]  Don’t know [ ] |  |
| --- | --- | --- | --- |
| Q41 | I consider owning and always sleeping under a mosquito net is very important. | Yes [ ]  No [ ]  Not sure [ ]  Don’t know [ ] |  |
| Q41 | I think it is necessary to use a treated mosquito net to prevent malaria. | Yes [ ]  No [ ]  Not sure [ ]  Don’t know [ ] |  |
| Q42 | My household is affected by malaria | Yes [ ]  No [ ]  Not sure [ ]  Don’t know [ ] |  |
| Q43 | Malaria is a matter of concern for me and my children. | Yes [ ]  No [ ]  Not sure [ ]  Don’t know [ ] |  |
|  | Malaria can have long-term consequences for my health or that of my children. | Yes [ ]  No [ ]  Not sure [ ]  Don’t know [ ] |  |
|  | At what time of day do you think mosquitoes bite the most? | Daytime…………………………….[ ]  Night time [ ]  Any time [ ]  Don’t know [ ] |  |
|  | Is it important to have a test before taking malaria treatment? | Yes [ ]  No [ ]  Not sure [ ]  Don’t know [ ] |  |
|  | What do you consider to be the best cure for malaria? | Paracetamol………………………...[ ]  Coartem…………………………….[ ]  Traditional/Herbal medications…….[ ]  Don’t know [ ] |  |

**Section E: Practices towards malaria Prevention**

| Q55 | **Do you do anything to protect yourself and your household members from malaria?**  SINGLE RESPONSE | Yes [ ]  No [ ] |  |
| --- | --- | --- | --- |
| Q56 | **What do you do?**  MULTIPLE RESPONSES POSSIBLE  CIRCLE ALL MENTIONED  PROBE ONCE: ANYTHING ELSE? | Sleep under a mosquito bed net [ ]  Use netting on windows [ ]  Try not to be outside during night-time [ ]  Use insecticide spray [ ]  Wearing protective clothing [ ]  Use mosquito repellent [ ]  Clearing bushes around the house [ ]  Getting rid of stagnant water [ ]  Regular clean-ups around the house [ ]  Other Specify:_______________________ |  |
| Q45 | **Do you and other members of the household sleep under insecticide treated bed nets?** | Yes ……………………………………….[__]  No ………………………………………..[__] |  |
|  | **If Yes to Q45, When do you sleep under the mosquito nets?** | Always ………………………….………..[__]  Yes, but only when it’s cold ……………..[__]  Yes, but only in the rainy season ……...…[__]  Only when I feel like ………………….....[__]  When I see mosquitos in the house ……...[__]  Other Specify __________________________ |  |
| Q46 | **If No to Q44, Why not?** | It has not been distributed in our area…….[__]  I don’t like sleeping under a mosquito net..[__]  I haven’t collected it yet…………………..[__]  Don’t know what I can do …………. [ ]  I’m not concerned about malaria [ ]  I don’t have money [ ]  It is not practical [ ]  Other Specify __________________________ |  |
| Q47 | **Did all the under-five years children sleep under a mosquito net during the previous night?** | Yes………………………………………[__]  No ………………………………………[__] |  |
| Q48 | **What do you do when you notice any sign or symptom of malaria for your child?** | Get medication he/she didn’t finish the last time he/she had Malaria……………………….[__]  Take herbs ………………………………[__]  Go get drugs from a pharmacy around…..[__]  Take paracetamol/Eferegan …………...... [__]  Wait to see if the situation will change…..[__]  Go to the hospital………………………...[__] |  |
| Q49 | **Do you request for a malaria test before treating your child?** | Yes ………………………………………[__]  No ……………………………………….[__] |  |
| Q50 | **If Yes why?** | To be sure that it is malaria ……………..[__]  I like doing the test ……………………...[__]  I don’t mind doing it …………………….[__]  Other Specify __________________________ |  |
| **Q51** | **If no why?** | I know malaria symptoms so well that I don’t need a test ……………………………….[__]  It is expensive …………………………...[__]  It is painful ……………………………....[__]  I don’t like doing it ……………...…...….[__]  It will waste time ………………………..[__]  Other Specify __________________________ |  |
| **Q52** | **When given malaria drugs, how do you give them to your sick child?** | As prescribed by the health professional  When I remember  When I feel like  Others Specify _______________________ |  |
| **Q53** | **How long does your take the drugs?** | I respect the number of days prescribed by the health professional ……………………..…[__]  I take till it finishes ……………………….[__]  I stop when I feel better……………….......[__]  I don’t even take them ……………………[__]  Other Specify ________________________ |  |
